# The DNA Methylation–Autophagy Axis: A Driver of MSC Fate Imbalance in Skeletal Aging and Osteoporosis

**DOI:** 10.3390/biology15030218

**Published:** 2026-01-24

**Authors:** Gaojie Song, Xingnuan Li, Jianjun Xiong, Lingling Cheng

**Affiliations:** 1 Jiangxi Provincial Key Laboratory of Cell Precision Therapy, School of Basic Medical Sciences, Jiujiang University, Jiujiang 332005, China; songgaojie163@163.com (G.S.);; 2 College of Veterinary Medicine, Gansu Agricultural University, Lanzhou 730070, China

**Keywords:** DNA methylation, autophagy, MSCs, aging, osteoporosis

## Abstract

Age-related osteoporosis is driven in part by senescence-associated rewiring of bone marrow mesenchymal stem cells (MSCs) from osteogenic toward adipogenic fates. Accumulating evidence indicates that epigenetic drift and reduced autophagy are not isolated lesions but are mechanistically coupled through a bidirectional DNA methylation and autophagy axis. Here, we summarize how promoter hypermethylation of genes involved in autophagy and osteogenesis suppresses autophagic flux and osteoblast lineage transcriptional programs. Conversely, autophagy insufficiency reshapes the methylome by limiting methyl donor availability, most notably S-adenosylmethionine (SAM), and by reducing the turnover of key epigenetic regulators, including DNA methyltransferases (DNMTs), ten-eleven translocation (TET) dioxygenases, and histone deacetylases (HDACs). This self-reinforcing circuitry exacerbates mitochondrial dysfunction, oxidative stress, and inflammation driven by the senescence-associated secretory phenotype (SASP), thereby stabilizing adipogenic bias and progressively impairing marrow niche homeostasis and bone remodeling. We further discuss therapeutic strategies to restore balance within this axis, including selective modulation of epigenetic enzymes; activation of AMP-activated protein kinase (AMPK) and mechanistic target of rapamycin (mTOR) signaling with downstream engagement of Unc-51-like autophagy-activating kinase 1 (ULK1) and transcription factor EB (TFEB); targeting sirtuin pathways; mitochondria- and autophagy-supportive natural compounds; and bone-targeted delivery approaches or rational combination regimens.

## Simple Summary

With aging, the differentiation potential of bone marrow mesenchymal stem cells (MSCs) shifts from osteogenic (bone-forming) to adipogenic (fat-forming) fates, contributing to the development of osteoporosis. This transformation is governed by two key processes: DNA methylation, which regulates gene expression by controlling whether genes are turned on or off, and autophagy, the cell’s waste disposal system that maintains cellular quality. In this review, we explain how DNA methylation and autophagy interact in a bidirectional feedback loop. Excessive DNA methylation can silence genes essential for autophagic function and osteogenesis, while compromised autophagic activity leads to metabolic disruption, resulting in the accumulation of epigenetic regulators that further exacerbate DNA methylation. This vicious cycle increases oxidative stress and inflammation, accelerating bone loss. Understanding this “methylation–autophagy axis” opens new therapeutic avenues, including the use of epigenetic modulators, metabolic and autophagy activators (e.g., targeting AMP-activated protein kinase (AMPK)–mechanistic target of rapamycin (mTOR)–transcription factor EB (TFEB) signaling), and bone-targeted drug delivery systems or combination therapies.

## 1. Introduction

Osteoporosis is the most prevalent metabolic bone disorder in the aging population worldwide and fundamentally arises from a disruption of the balance between bone resorption and bone formation [1]. Accumulating evidence indicates that senescence of marrow mesenchymal stem cells (MSCs) and a shift in their differentiation potential constitute a central cellular mechanism driving bone loss. With advancing age, MSCs progressively drift from an osteogenic toward an adipogenic fate, resulting in impaired bone formation and excessive marrow fat accumulation [2].

**Figure 1:**
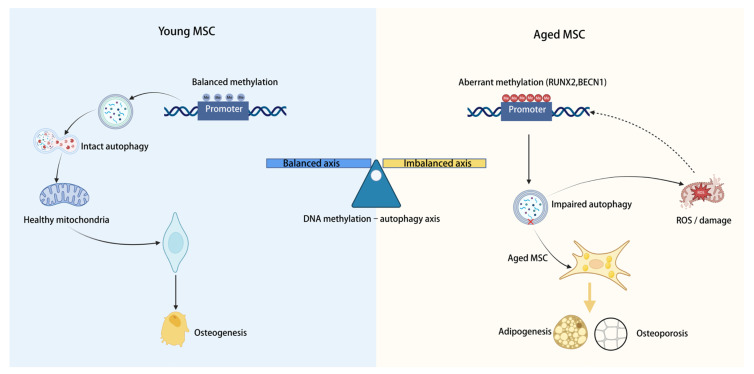
The DNA methylation–autophagy axis in young versus aged mesenchymal stromal cells (MSCs). In young or early-passage MSCs (**left**), competent autophagic flux sustains mitochondrial integrity and low reactive oxygen species (ROS) levels, thereby facilitating osteogenic differentiation. Promoters of autophagy- and osteogenesis-related genes (for example, runt-related transcription factor 2 (RUNX2) and Beclin 1 (BECN1)) remain hypomethylated and transcriptionally permissive, maintaining the DNA methylation–autophagy axis in a “balanced” state that favors bone formation. In aged MSCs (**right**), aberrant promoter hypermethylation of autophagy/osteogenic genes, impaired autophagy, and mitochondrial damage promote ROS accumulation. This “imbalanced” axis stabilizes adipogenic commitment at the expense of osteogenesis, ultimately contributing to trabecular bone loss and osteoporosis.

The differentiation fate of MSCs is governed by multilayered regulatory networks, among which autophagy and epigenetic modification emerge as two pivotal axes. Autophagy, a lysosome-dependent degradation pathway essential for cellular quality control, plays a key role in shaping cell fate, energy metabolism, and tissue homeostasis [3]. Decline of autophagic capacity in MSCs has been implicated as a critical determinant of age-associated impairment in osteogenic potential. However, much of the literature has centered on individual signaling cascades or lineage transcription factors—such as runt-related transcription factor 2 (RUNX2) and peroxisome proliferator-activated receptor γ (PPARγ)—with comparatively less emphasis on upstream, integrative regulatory modules [4,5,6]. Parallel advances in epigenetics have uncovered deeper layers of control underlying MSC aging. DNA methylation, by modulating promoter activity of osteogenic and adipogenic genes, acts as a major determinant of lineage specification [5,7]. At the same time, autophagy and DNA methylation are increasingly recognized as interconnected rather than independent events, influencing one another through metabolic pathways and the activity of epigenetic enzymes [8]. As illustrated in Figure 1, we propose the concept of a “DNA methylation–autophagy axis”, whereby metabolic and epigenetic crosstalk jointly establishes the fate equilibrium of MSCs and thereby drives aging-related bone loss [9]. In this review, we integrate current evidence on the molecular architecture, pathological consequences, and therapeutic implications of this axis, aiming to provide a refined conceptual framework for the prevention and treatment of osteoporosis.

## 2. The DNA Methylation–Autophagy Axis: An Integrative Bidirectional Circuitry

The “methylation–autophagy axis” refers to an integrated network of reciprocal interactions between DNA methylation and autophagy. This axis coordinates cellular energy metabolism, gene expression programs, and lineage decisions while offering a new molecular framework for understanding MSC aging. When dysregulated, it can generate self-reinforcing positive feedback loops that drive a functional shift in MSCs from osteogenic toward adipogenic lineages. In the following sections, we first discuss how DNA methylation acts as an upstream regulator of autophagy and then examine how autophagy in turn sculpts the epigenetic landscape.

### 2.1. Upstream Control of Autophagy by DNA Methylation

DNA methylation is catalyzed by DNA methyltransferases (DNMTs) and reversed by ten–eleven translocation (TET) family dioxygenases; the dynamic balance between these enzymes is essential for preserving MSC function [10,11]. During aging, global DNA methylation levels in MSCs tend to decline, yet selected regulatory loci become aberrantly hypermethylated, providing an important molecular basis for impaired autophagic capacity [12].

At the transcriptional level, DNA methylation can directly restrict the autophagy program. In aged MSCs, promoter regions of core autophagy genes—including Beclin 1 (BECN1), autophagy-related 5 (ATG5), and microtubule-associated protein 1A/1B-light chain 3 (LC3)—acquire repressive features such as hypermethylation and hypoacetylation, which associate with reduced transcription and compromised autophagic flux [13,14]. Transcription factor EB (TFEB) is a master transcriptional regulator of lysosomal biogenesis and the autophagy–lysosome pathway. In hepatocytes and renal tubular epithelial cells, promoter DNA methylation has been reported to repress TFEB expression [15,16]. In MSCs, a similar epigenetic constraint may contribute to a blunted stress-induced autophagic response. This coordinated regulation of both autophagy effectors and master regulators positions DNA methylation as a key upstream determinant that limits autophagy–lysosome transcriptional capacity, modulating the extent to which autophagy can respond to metabolic stress and regulate lineage-specific differentiation.

DNA methylation also shapes MSC lineage allocation by modulating transcription factors and signaling pathways that couple autophagy to differentiation. For instance, demethylation of the RUNX2 promoter is a critical early step in initiating osteogenic programs, whereas aging or oxidative stress can drive remethylation and silencing of this region, suppressing the transcription of osteogenic genes [17,18]. These findings highlight the dynamic nature of epigenetic control and its capacity to integrate autophagic status with differentiation cues.

Beyond canonical autophagy and osteogenic genes, DNA methylation targets transcription factors that coordinate stress responses and autophagic competence. Age-associated hypermethylation of the forkhead box O3 (FOXO3) promoter by DNA methyltransferase 3A (DNMT3A) directly represses FOXO3 transcription, weakening downstream antioxidant defenses and autophagy activation and thereby aggravating MSC senescence and osteoporotic phenotypes. Conversely, genetic or pharmacological attenuation of DNMT3A in animal models restores FOXO3 expression and significantly alleviates osteoporosis-related bone loss [19,20], underscoring DNMT3A as a druggable upstream node within this regulatory axis.

Taken together, DNA methylation governs autophagy in MSCs through direct transcriptional control of autophagy/autophagy–lysosome genes and methylation-sensitive tuning of autophagy-relevant signaling nodes that couple stress and fate cues to the autophagy machinery, including AMP-activated protein kinase (AMPK)–the mechanistic target of rapamycin complex 1 (mTORC1) regulation of the Unc-51-like autophagy-activating kinase 1 (ULK1)/TFEB axis and FOXO3-centered stress-response signaling. Excessive or misdirected methylation of key loci can repress autophagy-related transcription, erode cellular homeostasis, and bias MSCs toward an adipogenic fate.

### 2.2. Core Functions of Autophagy in Epigenetic Regulation

Autophagy is not merely a downstream target of epigenetic regulation; it can actively remodel the cellular epigenetic landscape. This influence is mediated through three tightly coupled routes: maintaining methyl-donor metabolism, enabling selective turnover of epigenetic enzymes, and integrating nutrient/energy sensing through the sirtuin family (SIRTs)–AMPK–TFEB module. Through these mechanisms, autophagy can act upstream of chromatin state and transcriptional output. Below, we discuss these routes in terms of metabolic substrate availability, proteostatic control of epigenetic regulators, and signaling architecture.

#### 2.2.1. Metabolic Control of Epigenetics: Autophagy Maintains Methyl-Donor Homeostasis

Autophagy is tightly coupled to one-carbon metabolism and is essential for maintaining the pool of methyl donors required for DNA and histone methylation [21,22,23]. S-adenosylmethionine (SAM), the principal methyl donor for these reactions, is generated through the coordinated activity of mitochondrial metabolism and the one-carbon cycle [24,25]. By clearing damaged mitochondria and recycling amino acids, autophagy sustains mitochondrial function and supports SAM biosynthesis [26]. Evidence from cancer and metabolic disease models suggests that reduced autophagic flux can disrupt cellular energy metabolism, limit SAM biosynthesis, and thereby decrease methyl-donor availability for DNA methyltransferases, reshaping DNA methylation programs [27]. Although this metabolism–epigenetics axis has been supported in other systems, its direct mechanistic contribution in senescent MSCs remains unresolved and warrants focused investigation.

Further studies indicate that impaired autophagy can in turn destabilize the activity of enzymes within the one-carbon metabolic network, thereby reinforcing a vicious cycle between metabolic stress and epigenetic dysregulation. In aged MSCs, reduced autophagic flux is accompanied by weakening of the folate cycle and related one-carbon pathways, together with diminished capacity to generate methyl donors, which may aggravate global and locus-specific DNA methylation imbalance [14]. Thus, by preserving the availability of metabolic substrates, autophagy provides both the energetic and material basis for proper epigenetic control. This coupling between metabolism and epigenetics is inherently bidirectional and dynamic. Mitochondrial deacetylases, exemplified by SIRT3, participate in this fine-tuning network. Sirtuin 3 (SIRT3) preserves mitochondrial integrity by deacetylating key substrates such as superoxide dismutase 2 (SOD2) at Lys68, thereby restraining ROS accumulation and sustaining adenosine triphosphate (ATP) production. With advancing age, SIRT3 expression declines, resulting in excessive ROS, impaired bioenergetics, and secondary suppression of autophagy and osteogenic differentiation. Conversely, enhancing SIRT3 expression in aged MSCs restores mitochondrial quality control, attenuates oxidative stress, and revitalizes their osteogenic potential [28,29].

#### 2.2.2. Epigenetic Remodeling Through Protein Degradation: Autophagy Shapes the Epigenetic Enzyme Landscape

Beyond its metabolic roles, autophagy directly remodels the epigenetic landscape via selective degradation of chromatin-modifying enzymes. The autophagy receptor p62/SQSTM1 has been validated across multiple cell types as a selective cargo receptor that recognizes diverse protein substrates and delivers them to lysosomes for degradation [30,31,32]. In cancer cells, senescent fibroblasts and myogenic cells, p62/SQSTM1, have been shown to target epigenetic regulators for lysosomal turnover, including DNA methyltransferase 1 (DNMT1), members of the TET family, and histone deacetylases (HDACs) [33,34,35]. Under physiological conditions, this mechanism helps to clear aged, damaged, or aberrantly aggregated epigenetic factors, thereby preserving a flexible chromatin configuration. In contrast, autophagic dysfunction in aged MSCs permits abnormal accumulation of DNMT1 and related enzymes that reinforce a rigid, hypermethylated state. As a consequence, osteogenic genes such as RUNX2 and alkaline phosphatase (ALP) remain persistently silenced by promoter hypermethylation, whereas adipogenic genes including PPARγ become aberrantly activated through demethylation [36,37]. This enzyme-driven “locking” of chromatin states is a major epigenetic force behind MSC fate drift.

The interplay between autophagy and the epigenetic machinery is further exemplified by histone deacetylase 6 (HDAC6), which serves as both a potential autophagic cargo and an active regulator of the autophagic process itself [38,39]. Under basal conditions, HDAC6 promotes autophagosome–lysosome fusion by deacetylating tubulin and recruiting ubiquitinated substrates to dynein motors. Under basal conditions, HDAC6 promotes autophagosome–lysosome fusion by deacetylating acetyl-α-tubulin (Lys40) and recruiting ubiquitinated substrates to dynein motors. Pharmacological inhibition of HDAC6 can restore microtubule integrity, increase acetyl-α-tubulin (Lys40), and improve autophagy function, emphasizing that its activity must be stringently controlled [40]. These observations indicate that feedback loops formed by “degradation of epigenetic enzymes” and “epigenetic regulation of autophagy components” jointly fine-tune cellular fate decisions.

Selective degradation of epigenetic modifiers by autophagy, and the regulation of autophagy by those same modifiers, are both subject to upstream control by nutrient- and lysosome-centered signaling pathways. The mTORC1–TFEB axis is particularly critical. When nutrients are abundant, activated mTORC1 phosphorylates TFEB (e.g., Ser142 and Ser211) and retains it in the cytoplasm, thereby repressing its transcriptional program [41,42]. Under conditions of nutrient deprivation or oxidative stress, mTORC1 activity is inhibited, allowing TFEB dephosphorylation at these sites (e.g., Ser142/Ser211) and nuclear translocation, where it acts as a master regulator to induce genes involved in autophagy and lysosomal biogenesis [43,44], consistent with residue-level mapping of TFEB phosphorylation/dephosphorylation [42]. This dynamic switch positions autophagy as a central bridge between metabolic cues and epigenetic reprogramming.

#### 2.2.3. A Signaling Hub for Integration: Bidirectional Coupling Within the SIRT–AMPK–TFEB Network

Recent work suggests that the role of autophagy in epigenetic regulation relies on tight integration between energy sensing and chromatin modification. Seminal studies in non-MSC systems, including neurons and cancer cells, identified a TIP60–ULK1 signaling axis in which the histone acetyltransferase TIP60 acetylates the autophagy-initiating kinase ULK1 (reported at Lys162 and Lys606), thereby revealing an acetylation-dependent layer of control over autophagy initiation [45,46]. AMPK, activated in response to energy depletion, directly phosphorylates ULK1 (e.g., Ser317 and Ser777) and antagonizes mTOR signaling, thereby promoting autophagic flux [47,48]. In parallel, the deacetylase SIRT1 reshapes the DNA methylation environment by deacetylating DNMT1 and HDAC1, and facilitates TFEB deacetylation (e.g., Lys116) and nuclear import, which enhances transcription of autophagy- and lysosome-related genes [42,49]. Through this dual control of chromatin modifiers and master transcription factors, SIRT1 links cellular energy status to epigenetic remodeling and autophagy induction.

During MSC aging, impaired nutrient/energy sensing is often accompanied by reduced TFEB nuclear activity and a weakened autophagy–lysosome program, changes that together erode osteogenic competence. In aged bone-marrow MSCs, pharmacological enhancement of TFEB-driven autophagy restored autophagic flux and improved osteogenic output in vitro, and attenuated age-associated bone loss in middle-aged mice in vivo [50]. In parallel, mechanical loading/exercise has been reported to increase autophagy in primary bone marrow mesenchymal stem cells (BMSCs) in a SIRT1-dependent manner and to promote osteogenic differentiation in vitro, linking pro-osteogenic cues to autophagic remodeling in an aging context [51]. In vitro, primary jawbone-derived BMSCs with reduced sirtuin 6 (SIRT6) exhibited diminished autophagy activity together with impaired osteogenic differentiation; in vivo, SIRT6 insufficiency was associated with age-dependent jawbone loss. Mechanistic analyses in the same study further implicated protein kinase B (Akt)–mTOR signaling as an upstream regulator of autophagy in this craniofacial MSC context [52]. Taken together, these observations support a model in which the sirtuin–AMPK–TFEB axis integrates metabolic state with autophagy–lysosome activity to shape osteogenic fate decisions in MSCs while also highlighting the importance of clearly defining cell source and experimental context when interpreting this bidirectional coupling. Representative PTM sites discussed in this signaling hub are summarized in Supplementary Table S1.

#### 2.2.4. Summary: Formation of the Autophagy–Methylation Feedback Loop

**Figure 2:**
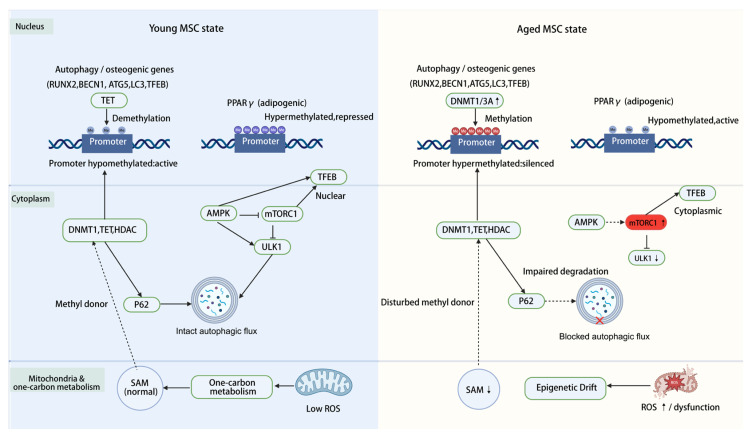
Molecular circuitry linking DNA methylation, autophagy, and one-carbon metabolism in young and aged MSCs. Black arrows indicate promotion/activation, while ‘T’ bars denote inhibition/suppression. (**Left**): In bone marrow-derived MSCs from young donors or early-passage cultures, promoters of autophagy and osteogenesis-related genes, including RUNX2, BECN1, autophagy-related protein 5 (ATG5), microtubule-associated protein 1 light chain 3 (LC3), and transcription factor EB (TFEB), are hypomethylated and transcriptionally active. Ten-eleven translocation (TET)-mediated demethylation and adequate S-adenosylmethionine (SAM) supply from intact mitochondrial and one-carbon metabolism maintain an open chromatin state. In the cytoplasm, AMP-activated protein kinase (AMPK) inhibits mechanistic target of rapamycin complex 1 (mTORC1), activates unc-51 like autophagy activating kinase 1 (ULK1), and enhances nuclear TFEB, sustaining autophagic flux. p62-mediated degradation of excess DNA methyltransferases (DNMTs), TETs, and histone deacetylases (HDACs) further prevents accumulation of epigenetic enzymes. (**Right**): In aged MSCs, increased DNMT1/3A activity drives promoter hypermethylation and silencing of autophagy and osteogenesis genes, whereas peroxisome proliferator-activated receptor gamma (PPARγ) remains hypomethylated and active. AMPK is suppressed, and mTORC1 is overactivated, leading to ULK1 inhibition, TFEB retention in the cytoplasm, and blocked autophagic flux. Impaired p62-dependent degradation of DNMTs, TETs, and HDACs, along with decreased SAM production (“disturbed methyl donor”), reinforces epigenetic drift. Mitochondrial dysfunction and ROS accumulation further exacerbate this vicious cycle.

In summary, autophagy sustains the supply of methylation substrates and regulates the abundance and activity of epigenetic enzymes, forming a bidirectional regulatory loop with DNA methylation. While DNA methylation sets the transcriptional potential of the autophagy machinery, autophagy reciprocally influences methylation reactions by controlling methyl-donor availability and the balance of methyltransferases and demethylases. In the context of aging, amplification of this feedback loop traps MSCs in a state marked by epigenetic abnormalities, elevated oxidative stress, and metabolic disequilibrium, driving a gradual shift from osteogenic toward adipogenic lineage commitment. The major molecular players involved in the DNA methylation–autophagy axis are summarized in Table 1. The spatial and temporal integration of these molecular events, schematically illustrated in Figure 2, provides a unifying mechanistic backdrop for the subsequent discussion of MSC fate remodeling and age-related bone loss.

## 3. Lineage Fate Regulation: Pathological Consequences of an Imbalanced DNA Methylation–Autophagy Axis

Building on the autophagy–methylation feedback loop described above, this section focuses on how disruption of this axis is amplified at the levels of lineage fate and the bone marrow microenvironment. Functional imbalance of the DNA methylation–autophagy axis is a hallmark that spans the entire course of MSC aging. Its central pathological outcome is a reprogramming of lineage fate: MSCs drift from an osteogenic toward an adipogenic trajectory, leading to diminished osteogenic capacity and increased adipogenesis. In turn, these cell-intrinsic changes drive bone loss and disturb marrow homeostasis, forming the molecular basis of age-related osteoporosis.

### 3.1. Locking Lineage Fate: Imbalance of DNA Methylation and Autophagy

In bone marrow-derived MSCs from young donors or early-passage cultures, the chromatin surrounding osteogenic master transcription factors such as RUNX2 and its cofactor Osterix (SP7) is in an open, transcriptionally permissive state. Their promoters display low levels of DNA methylation, autophagic flux is robust, and metabolic activity is sufficient to support matrix production, together conferring strong osteogenic potential [55,61]. With aging and the associated rise in oxidative stress, DNMTs become aberrantly recruited to the promoters of these osteogenic genes, driving hypermethylation and transcriptional silencing. As a result, the “core switches” that initiate osteogenic programs are chronically switched off [37].

In parallel, promoters of adipogenic drivers including PPARγ and CCAAT/enhancer-binding protein α (C/EBPα) become progressively demethylated during aging. This results in their sustained activation and irreversibly commits MSCs toward adipocyte differentiation [56,62]. This reciprocal pattern of “osteogenic silencing and adipogenic activation” establishes a lineage-locking effect. Consequently, even when exogenous osteoanabolic cues such as bone morphogenetic protein 2 (BMP2) or Wingless/Integrated (Wnt) ligands are supplied, the rigidified chromatin architecture prevents complete reactivation of the osteogenic transcriptional program. Autophagy defects in aged MSCs further exacerbate this situation by allowing abnormal accumulation of DNMT1, HDACs, and other epigenetic modifiers, which reinforce the high-methylation, low-autophagy loop [18]. The net result is persistent repression of osteogenic genes and a permissive epigenetic landscape for adipogenic programs, establishing a molecular foundation for lineage fate bias.

### 3.2. Fine Sculpting by Histone Modifications, Non-Coding RNAs, and Nuclear Architecture

Beyond DNA methylation, histone modifications and non-coding RNAs compose an additional layer of epigenetic regulation that shapes MSC aging and lineage choice. These regulators intersect with the methylation–autophagy axis and jointly stabilize age-associated shifts in differentiation potential.

In addition to covalent chromatin marks, nuclear-lamina organization provides a nuclear-architecture-based epigenetic layer that can bias mesenchymal stem/progenitor fate decisions. Lamins and inner-nuclear-membrane scaffolds organize lamina-associated domains (LADs), which define a transcriptionally repressive nuclear-peripheral chromatin environment enriched for gene-silencing complexes and thereby constrain lineage-inappropriate programs [63]. In skeletal stem cells (SSCs), extracellular matrix (ECM) remodeling modulates the abundance of nuclear lamin A/C and the localization of emerin, thereby regulating β-catenin/Wnt signaling and influencing the balance between osteogenic and adipogenic differentiation both in vitro and in vivo [64]. Complementarily, in primary human bone marrow mesenchymal stem/stromal cells (hBMSCs), topographical cues increased lamin A/C and were accompanied by locus-level reprogramming of osteogenic chromatin (e.g., reduced trimethylation of histone H3 lysine 27 (H3K27me3) and increased acetylation of histone H3 lysine 9 (H3K9ac) at ALPL/RUNX2/OCN-associated loci), supporting a lamina–chromatin axis upstream of osteogenic gene activation [65]. Consistent with a role in stem-cell aging, LMNA-mutant induced pluripotent stem cell (iPSC)-derived mesenchymal lineages (including MSCs) exhibit LAD reorganization, disordered chromatin hierarchy, and accelerated senescence, highlighting nuclear lamina integrity as a convergent node linking chromatin state and mesenchymal stem-cell dysfunction [66].

At the histone level, histone deacetylase 9 (HDAC9) has emerged as a critical mediator of this pathological transition. HDAC9 expression is markedly increased in aged MSCs. By deacetylating histones at pro-autophagic gene loci, HDAC9 represses autophagy gene expression while simultaneously amplifying tumor protein p53 (p53)-driven senescence signaling. Inhibition of HDAC9 in aged MSCs reactivates autophagic flux, attenuates senescence pathways, and partially restores the balance between osteogenic and adipogenic differentiation. Consistent with these cellular observations, silencing HDAC9 in aged mice enhances bone formation, reduces marrow fat accumulation, and improves bone mineral density [54], establishing HDAC9 as an important “epigenetic brake” on osteogenesis in the aging skeleton.

At the level of noncoding RNAs, studies in human and mouse bone MSCs indicate that age-associated dysregulation of multiple microRNAs (miRNAs) can bias lineage commitment. For example, microRNA-188 (miR-188) is markedly upregulated in aged bone marrow and MSCs, where it promotes adipogenic differentiation by directly targeting the pro-osteogenic regulators rapamycin-insensitive companion of mTOR (RICTOR) and histone deacetylase 9 (HDAC9); in vivo inhibition of miR-188 can attenuate age-related bone loss [67]. During human MSC senescence, miR-34a increases, and miR-34a inhibition has been shown to alleviate senescence-associated phenotypes, at least in part by restoring or activating SIRT1 [68]. Mechanistically, miR-34a-mediated repression of SIRT1 enhances p53 acetylation and activity, which in turn induces miR-34a, forming a p53–miR-34a–SIRT1 positive feedback loop [69]. In osteoporotic bone tissue and MSCs, miR-15b is upregulated and has been linked to suppressed autophagy and osteogenic differentiation through targeting the deubiquitinase ubiquitin-specific protease 7 (USP7) [70].

Long noncoding RNAs (lncRNAs) further expand and rewire this regulatory network. In aged mouse MSCs, lncRNA NEAT1 is markedly increased and has been associated with mitochondrial dysfunction and disruption of pluripotency-associated networks, skewing differentiation toward adipogenesis; Neat1 knockdown in aged mice reduces marrow adiposity and increases bone mass [71]. By contrast, in osteoporosis models, lncRNA small nucleolar RNA host gene 14 (SNHG14) acts as a competing endogenous RNA (ceRNA) to sequester miR-493-5p, thereby de-repressing targets such as myocyte enhancer factor 2C (MEF2C), activating autophagy and enhancing osteogenic differentiation, which may slow disease progression [72]. In human bone marrow MSCs, the maternally expressed lncRNA H19 and its derivative miR-675 can relieve epigenetic repression of osteogenic genes by downregulating class II HDACs, thereby promoting osteogenesis while restraining adipogenesis [73].

Taken together, histone-modifying enzymes and non-coding RNAs are tightly integrated with the DNA methylation–autophagy axis (summarized in Table 2). Through multilayered epigenetic reprogramming, they act in concert to finely control and ultimately stabilize the trajectory of MSC fate during aging.

### 3.3. The Metabolism–Autophagy–Fate Triangle: Reshaping Lineage Decisions by Energy Status

Commitment of MSCs to the osteogenic lineage is an energy-intensive process that requires robust synthesis, secretion, and mineralization of extracellular matrix proteins [74,75]. Autophagy supports this process by recycling amino acids, maintaining mitochondrial function, and providing ATP and biosynthetic precursors [53,76,77]. When autophagy is suppressed, damaged organelles accumulate, metabolic flux stalls, and ROS levels rise, ultimately leading to mitochondrial dysfunction and reduced ATP production [78,79]. Accordingly, dysfunction of the AMPK–mTOR–TFEB module (mechanistic details in Section 2.2.3) constrains the metabolic capacity required for osteogenic differentiation. Conversely, consistent with this module’s role in sustaining autophagic capacity, AMPK-activating interventions (e.g., metformin) can enhance autophagic flux and restore the mineralization capacity of MSCs [57,80,81,82], providing strong support for a causal coupling between metabolism, autophagy, and lineage fate.

Concomitantly, autophagy deficiency disrupts lipid homeostasis, leading to lipid droplet accumulation and impaired fatty acid oxidation. The ensuing oxidative stress (a consequence of both mitochondrial dysfunction and metabolic shift) activates PPARγ and p38 MAPK signaling, which further promote adipogenic commitment while suppressing osteogenic programs [58,83,84,85]. Thus, autophagy serves as a metabolic gatekeeper, and its impairment shifts MSC fate toward lipid storage and away from bone formation.

Following osteogenic commitment of MSCs, osteoblast differentiation and maturation are executed as a staged transcriptional program governed by coordinated transcriptional and signaling networks, rather than a single RUNX2-driven switch [86,87]. Consistent with this staged architecture, single-cell atlases of mouse and human osteoblast-lineage cells delineate discrete maturation states that range from RUNX2/SP7-enriched early osteoblasts and matrix-producing populations (e.g., COL1A1/2, ALPL) to more mature mineralizing states marked by higher expression of mineralization-associated genes, including integrin-binding sialoprotein (IBSP), secreted phosphoprotein 1 (SPP1), and bone gamma-carboxyglutamate protein/osteocalcin (BGLAP/OCN) [88,89,90]. Autophagy is likewise dynamic across differentiation: while basal flux can support early commitment and bioenergetic remodeling, sustained or excessive autophagy during late maturation can become inhibitory, particularly under lipid overload. In osteoblast-lineage cultures exposed to lipid excess, activation of lipophagy further suppressed mineralization, whereas partial autophagy inhibition modestly restored osteogenesis [91]. Consistently, in rapamycin-treated MC3T3-E1 cells, RUNX2 overexpression enhanced mineral deposition while dampening autophagy, supporting an inhibitory autophagy window during maturation and mineralization [92]. Collectively, these data support a stage- and metabolic-context-dependent relationship between autophagy (including lipophagy) and osteogenesis, arguing against a uniformly pro-osteogenic role of autophagy [93,94].

### 3.4. Inflammatory Niche Amplification of Autophagy–Epigenetic Imbalance

The aged marrow niche is characterized by chronic inflammation, which is further amplified by mitochondria-derived redox stress secondary to defective autophagy (see Section 3.3), thereby amplifying niche-level inflammatory remodeling. In immune cells and other somatic cell types, impaired autophagy can promote mitochondrial DNA (mtDNA) release into the cytosol, which activates the cGAS–STING (cyclic GMP–AMP synthase–stimulator of interferon genes) and nuclear factor kappa B (NF-κB) signaling pathways. This activation drives the production of inflammatory cytokines such as interleukin-6 (IL-6) and interleukin-1β (IL-1β) [59,60]. These mediators not only inhibit osteogenic differentiation of MSCs but also enhance osteoclast formation and activity, fueling a vicious cycle in which inflammation and bone resorption reinforce each other.

Notably, metabolic stressors can further exacerbate lipid peroxidation-driven injury (including ferroptotic features), compounding osteolineage attrition under aging-associated metabolic burden [95,96]. In parallel, senescent cells adopt a senescence-associated secretory phenotype (SASP), releasing pro-inflammatory cytokines, chemokines, and proteases that not only intensify local inflammation but also induce senescence in neighboring cells via paracrine signaling, thereby establishing a “senescence-inflammation-osteogenesis inhibition” loop [97,98].

Collectively, autophagy defects and epigenetic abnormalities cooperate to generate a bone marrow microenvironment that is pro-inflammatory, anti-osteogenic, and pro-adipogenic. At the tissue level, this milieu amplifies the consequences of methylation–autophagy axis imbalance and accelerates skeletal aging.

### 3.5. From Cellular Fate to Systemic Phenotype: Bone Loss and Marrow Adipose Infiltration

Under physiological conditions, MSCs rely on the coordinated activity of the DNA methylation–autophagy axis to maintain a dynamic balance between osteogenic and adipogenic differentiation, thereby preserving bone marrow homeostasis [18,99]. When this axis becomes dysfunctional, the epigenetic and metabolic networks of MSCs are globally rewired: osteogenic programs are chronically suppressed, whereas adipogenic signals remain persistently overactivated. Hypermethylation and silencing of RUNX2 and its downstream targets erode the ability of MSCs to differentiate into osteoprogenitors [75]. Simultaneously, autophagy defects interrupt energy supply and disrupt protein homeostasis, rendering key osteogenic signaling pathways, including Wnt/β-catenin and BMP/Smad, less responsive to anabolic cues [54,57].

**Figure 3:**
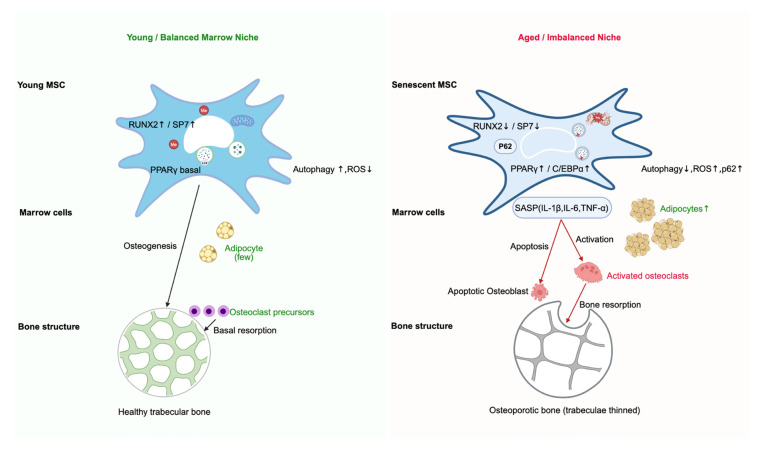
From MSC fate shift to marrow niche imbalance and osteoporotic bone loss. (**Left**): In the young and balanced marrow niche, MSCs exhibit high osteogenic potential with active RUNX2/SP7 programs and basal PPARγ activity. Autophagy is efficient, and ROS levels are low, supporting osteoblastogenesis with minimal adipogenesis. Trabecular bone is dense and well connected, and osteoclast activity is restricted to basal bone resorption. (**Right**): In the aged and imbalanced niche, senescent MSCs show suppressed RUNX2/SP7 activity and upregulated PPARγ/cCAAT/enhancer-binding protein alpha (C/EBPα), along with impaired autophagy, mitochondrial ROS accumulation, and p62 elevation. These cells acquire a senescence-associated secretory phenotype (SASP), characterized by elevated interleukin-1 beta (IL-1β), interleukin-6 (IL-6), and tumor necrosis factor-alpha (TNF-α), which promote osteoblast apoptosis, osteoclast activation, and expansion of marrow adipocytes. The combined effects lead to trabecular thinning, increased bone resorption, and osteoporotic bone architecture.

On the other side of the fate spectrum, adipogenic pathways remain active in a low-methylation, energy-storing environment, and PPARγ-driven feedback loops further suppress RUNX2 expression, making reversal of lineage drift increasingly difficult [100]. Chronic autophagy insufficiency promotes mitochondrial dysfunction and ROS accumulation, which in turn trigger inflammatory signaling and reinforce the senescent phenotype. This feeds into a “metabolic imbalance–epigenetic locking–inflammatory amplification” cycle. At the cellular level, this cycle consolidates adipogenic bias; at the microenvironmental level, SASP factors reshape the marrow niche, inhibit osteoblast differentiation, and promote osteoclast activation [101]. Consequently, MSCs progressively lose osteogenic competence, the dynamic balance between bone formation and resorption collapses, and bone mass declines, leading to osteoporosis and increased skeletal fragility. These multilevel changes converge within the bone marrow niche and bone architecture as hallmarks of skeletal aging, as schematically depicted in Figure 3. The translation of MSC fate bias into tissue- and organ-level pathology provides the pathological foundation for subsequent discussion of therapeutic strategies targeting the DNA methylation–autophagy axis.

## 4. Translational Prospects for Aging-Related Bone Disorders: Opportunities and Challenges

The concept of a DNA methylation–autophagy axis not only deepens our mechanistic understanding of MSC aging but also opens new avenues for intervention in aging-related bone disorders. In contrast to traditional single-pathway strategies, interventions centered on this axis can be broadly categorized into three complementary approaches: (1) targeting upstream epigenetic regulators to “reboot” chromatin programs by modulating DNA methylation and histone marks (Section 4.1); (2) restoring autophagic flux and mitochondrial homeostasis through metabolic reprogramming and bioactive small molecules, thereby correcting imbalance within the metabolism–autophagy–fate triangle (Section 4.2); and (3) combining pharmacological agents with bone-targeted delivery and lifestyle interventions such as nutrition and exercise to achieve system-level reconstruction from cells to the whole organism (Section 4.3). In the following subsections, we summarize the evidence base for these strategies and discuss their translational potential.

### 4.1. Upstream Epigenetic Targets: Resetting Chromatin and Correcting Fate Bias

At the epigenetic level, DNA methyltransferase inhibitors such as decitabine have been shown to reverse aberrant hypermethylation and silencing of key osteogenic genes, including RUNX2 and Osterix, thereby reactivating their transcription. In parallel, pharmacological activation of TET enzymes promotes active DNA demethylation and can enhance the osteogenic potential of MSCs [102,103]. However, these agents are frequently limited by substantial systemic toxicity and broad off-target effects, constraining their clinical utility. This has shifted recent efforts toward bone-targeted delivery systems. For example, nanoparticles decorated with bone-homing peptides can concentrate epigenetic drugs within the skeletal compartment, increasing local efficacy while markedly reducing systemic adverse effects [104]. A focused Spotlight on bone-targeted nanocarrier delivery is provided in Section 4.3.

Newer strategies aim to exploit aging-specific epigenetic alterations. DNMT3A-mediated hypermethylation of the FOXO3 promoter has been identified as a critical event driving redox imbalance and bone loss; knockdown of DNMT3A restores FOXO3 expression and attenuates skeletal loss in experimental models, underscoring DNMT3A as a high-value drug target [20].

The development of highly selective DNMT3A inhibitors or PROTAC-based degraders could prevent abnormal hypermethylation of FOXO3 and a subset of osteogenic gene promoters, thereby strengthening antioxidant defenses while reactivating osteogenic transcriptional programs.

Enhancing ten–eleven translocation 2 (TET2) activity represents another tractable intervention to reprogram aged MSCs. In osteoporotic animal models, augmenting TET2-dependent DNA demethylation has been reported to restore osteogenic gene expression and improve bone formation. In parallel, targeting histone methylation may reset the functional state of senescent MSCs [105]. In vitro, pharmacological inhibition of enhancer of zeste homolog 2 (EZH2) in MSCs isolated from osteoporotic bone reduces histone H3 lysine 27 trimethylation (H3K27me3), re-engages Wnt/β-catenin signaling, and promotes osteogenic differentiation [106]. In aged BMMSCs, histone deacetylase 9 (HDAC9) has emerged as a key epigenetic determinant of autophagy and lineage allocation. Small interfering RNA (siRNA)-mediated HDAC9 knockdown increases autophagic flux and rebalances osteogenic versus adipogenic differentiation in aged BMMSCs. In vivo, intramedullary delivery of a lentiviral vector encoding short hairpin RNA targeting HDAC9 (shHDAC9) partially rescues bone loss and improves the intrinsic osteogenic capacity of endogenous BMMSCs [54].

Histone acetylation dynamics play a crucial role in the axis linking methylation and autophagy. Histone acetyltransferases, including the Tat-interactive protein 60 (TIP60), modulate chromatin accessibility and contribute to autophagy initiation by acetylating key proteins, such as ULK1 [45,107]. Selective activation of TIP60, or inhibition of its antagonistic HDACs, may therefore allow simultaneous maintenance of autophagic competence and epigenetic remodeling. Overall, interventions targeting upstream epigenetic nodes provide powerful tools to “reset chromatin” and correct fate bias, but their success will depend on integration with bone-targeted delivery systems and careful dose optimization to ensure safety and specificity.

### 4.2. Metabolism–Autophagy Pathways and Natural Small Molecules: From Energy Reprogramming to Axis Repair

At the metabolic level, boosting autophagic flux has emerged as an effective strategy to ameliorate senescence-associated phenotypes in MSCs. Across multiple osteoporosis models, sirtuin deacetylases (sirtuins), via coupling to the AMPK–mTOR–ULK1 axis and mitochondrial deacetylation, coordinately tune autophagy, energy metabolism, and oxidative stress, thereby supporting bone mass and the osteogenic–adipogenic balance of MSCs [28]. Coordinated activation of SIRT1/AMPK and SIRT3 may help break the vicious cycle of autophagy deficiency and oxidative stress in aged MSCs, offering a new dimension for metabolic intervention in skeletal aging.

Within the canonical mTOR–autophagy cascade, rapamycin has been shown in bone marrow-derived mesenchymal stem cells (BM-MSCs) to relieve mTOR-dependent autophagy suppression and enhance osteogenic capacity, while MSC-focused in vivo models further link restoration of autophagic competence to attenuation of age-related skeletal deterioration [108,109]. Nonetheless, sustained and excessive autophagy may trigger apoptosis or functional exhaustion, emphasizing the need for precise control of dose and treatment timing [110]. Moreover, AMPK activators such as metformin may exert broader, pleiotropic control: beyond inducing autophagy via mTOR inhibition and direct ULK1 phosphorylation, metformin can concurrently improve energy metabolism and skeletal homeostasis. Accumulating preclinical and clinical evidence supports its favorable safety profile and translational promise [47,81,82,111]. Beyond these canonical pathways, strategies aimed at selectively activating TFEB—without inducing global mTOR inhibition—are gaining attention. By restoring both autophagy and mitochondrial homeostasis, TFEB-targeted interventions may provide a more precise means of mitigating skeletal aging [112,113,114].

Natural small molecules often modulate the methylation–autophagy axis by orchestrating a coupled metabolism–autophagy–redox–osteogenic signaling network, rather than acting through a single linear pathway. In aged MSCs in vitro, resveratrol engages AMPK while restraining PI3K/AKT/mTOR signaling, lowering ROS, stabilizing mitochondrial function, and promoting autophagy, with a net restoration of the osteogenic-to-adipogenic balance [115]. In placenta-derived MSCs, metformin likewise enhances osteogenic differentiation potential via AMPK activation [82]. In skeletal-relevant animal models, spermidine and urolithin A have been linked to induction of autophagy and mitophagy through engagement of the SIRT1/SOD2 program or modulation of PI3K/AKT/mTOR signaling, thereby alleviating oxidative injury and supporting mitochondrial homeostasis. Notably, spermidine increases bone mass in an iron overload-induced bone loss model alongside elevated SIRT1/SOD2 expression in bone tissue [116], whereas urolithin A shows bone-protective activity across multiple preclinical settings, at least in part by reinforcing mitochondrial quality control and autophagy competence [117]. Additional evidence comes from defined skeletal cell models. In bone marrow MSCs, quercetin promotes osteogenic differentiation and improves redox stress via Wnt/β-catenin and BMP2/Smad/RUNX2 signaling [118]. In MC3T3-E1 preosteoblasts, trifloroside enhances osteogenic and mineralization activity [119]. In C3H10T1/2 mesenchymal progenitor cells, melatonin enhances osteogenic differentiation by signaling through its specific receptors, melatonin receptor 1 (MT1) and melatonin receptor 2 (MT2) [119]. Collectively, these compounds converge on multiple nodes spanning redox defenses, AMPK-linked autophagy, and osteogenic pathways, biasing a dysregulated methylation–autophagy axis toward osteogenesis and away from adipogenesis, and supporting multi-target strategies for skeletal aging that remain compatible with long-term safety considerations.

### 4.3. Spotlight: Bone-Targeted Nanocarrier Delivery to Reprogram the DNA Methylation–Autophagy Axis

Bone-targeted nanocarrier systems provide a clinically relevant route to translate modulation of the DNA methylation–autophagy axis into skeletal-specific therapy by concentrating epigenetic or autophagy-regulating cargoes within the bone microenvironment while minimizing systemic exposure [120,121,122]. Using hydroxyapatite-binding ligands such as poly-aspartate peptides or bisphosphonates, these platforms can preferentially accumulate at bone remodeling surfaces and within the marrow niche—precisely where dysregulated MSC methylation programs and autophagy insufficiency drive adipogenic bias and osteogenic failure [123]. In preclinical osteoporosis models, targeted delivery has been associated with improved bone outcomes relative to untargeted approaches, consistent with restoration of osteogenic programs and autophagic competence in MSCs, while potentially offering a more favorable safety profile through reduced systemic exposure [124].

### 4.4. Combination Interventions and Integrated Prevention: From Single Targets to System Reconstruction

As our understanding of skeletal aging becomes more refined, combination therapy is progressively replacing traditional single-target approaches. An ideal therapeutic strategy likely involves two interconnected components: an “upstream reprogramming” element that reactivates silenced osteogenic gene programs through epigenetic drugs, metabolic modulators, or lifestyle interventions, and a “downstream protection” element that preserves cellular function and protects the osteogenic microenvironment through anti-inflammatory, antioxidant, and autophagy-enhancing measures. For example, in a type 2 diabetes-associated osteoporosis model, rosmarinic acid improves bone microarchitecture by inhibiting the TXNIP–NLRP3 inflammatory pathway, demonstrating the potential of combining epigenetic modulation with cellular protection within a single regimen [125]. Natural small molecules, such as quercetin and spermidine, are emerging as promising tools for low-toxicity, long-term interventions. These molecules exert effects through mechanisms such as AMPK activation, autophagy induction, mitochondrial protection, and epigenetic reprogramming. Lifestyle interventions, including regular aerobic exercise and caloric restriction, not only improve systemic metabolism and induce autophagy but also reshape the epigenetic landscape of the bone marrow stroma by altering DNA methylation and histone modifications. This creates a supportive context that may enhance and stabilize the effects of pharmacological therapies [126,127]. Looking forward, rational combinations of drugs, nutritional or natural compounds, and structured exercise programs may facilitate the construction of an integrated “metabolism–epigenetics–function” tri-dimensional strategy for skeletal aging. Such a systems-level framework could pave the way for more comprehensive and personalized management of aging-related bone diseases, with the DNA methylation–autophagy axis serving as a central organizing principle for intervention design.

## 5. Challenges and Perspectives

Although targeting the DNA methylation and autophagy axis is conceptually compelling, several barriers still limit clinical translation.

Biological breadth is the first constraint. DNA methyltransferases, autophagy programs, and metabolic control nodes operate across many tissues, so interventions placed too far upstream may improve MSC aging yet disturb extraskeletal homeostasis, with a meaningful oncogenic liability. This makes bone targeted and cell selective delivery foundational. Engineered extracellular vesicles, including exosomes, can be functionalized with bone marrow MSC-targeting peptides such as E7 to enrich uptake by marrow MSCs and sustain local exposure to nucleic acid therapeutics within the bone niche [128]. In parallel, siRNA enables sequence-guided, reversible silencing in vivo, providing a degree of controllability that systemic small molecule activation rarely achieves [129]. Together, these strategies refocus intervention from global pathway stimulation to niche repair.

Safety then becomes a problem of balance. Epigenetic modulation and autophagy manipulation are context dependent: excessive activation can destabilize genomes or trigger cytotoxic stress, whereas durable suppression can accelerate functional decline. Translation will therefore hinge on regimen design, including intermittent dosing and temporally controlled pathway engagement calibrated to restore homeostasis over time. Because MSC aging trajectories and lineage allocation vary by sex, disease context, and baseline biological age, stratification is likely to be required. Multidimensional aging scores integrating multiomics with minimally invasive sampling, including liquid biopsy, could provide quantitative readouts for patient selection and dosing.

Finally, progress will depend on systems control and translational practicality. Integrating artificial intelligence with multiomics should enable dynamic network models that couple autophagic flux to evolving methylation states, nominate regulatory bottlenecks, and define intervention windows with favorable benefit to risk profiles. In parallel, feasibility will be shaped by engineering and regulatory realities, including GMP compatible manufacturing, batch consistent cargo loading, validated potency assays, predictable biodistribution, immunogenicity risk, and long-term, off-target surveillance.

Overall, the DNA methylation and autophagy axis reframes skeletal aging intervention toward mechanism-informed precision strategies. The next step is not simply slowing decline but achieving controlled, partial rejuvenation of MSC function under stringent durability and safety constraints.

## 6. Conclusions

The DNA methylation–autophagy axis is a central determinant of MSC fate, coordinating osteogenic competence and adipogenic drift during skeletal aging. When promoter hypermethylation converges with impaired autophagic flux, MSCs become epigenetically constrained, metabolically stressed, and progressively biased toward adipogenesis, thereby accelerating bone loss. Therapeutic translation will likely require interventions that jointly recalibrate aberrant methylation programs and restore autophagy-linked organelle quality control, coupled with bone-targeted, cell-selective delivery to limit extraskeletal exposure. Targeting this self-reinforcing circuitry may thus shift intervention from downstream symptom control toward mechanism-informed restoration of niche homeostasis under stringent safety constraints.

## Figures and Tables

**Table 1:** Major molecular players in the DNA methylation–autophagy axis.

Module	Molecule(s)	Specific Role in the Axis (MSC Aging and Fate Imbalance)	References
DNA methylation (writers)	DNMT1	DNA methyltransferase that restrains osteogenic programs; autophagy-dependent DNMT1 turnover reported in other systems suggests that reduced flux may stabilize DNMT1 and reinforce hypermethylation.	[33,37]
DNA methylation (writers)	DNMT3A	DNMT3A-mediated hypermethylation of FOXO3 has been shown in osteoclast-lineage settings; FOXO3 supports BMMSC osteogenesis partly via autophagy, implicating a DNMT3A–FOXO3 stress/autophagy node.	[19,20]
DNA demethylation (erasers)	TET family (e.g., TET2)	TET dioxygenases sustain active DNA demethylation; autophagy-dependent TET2 degradation reported in a viral context links autophagy status to demethylation capacity.	[11,34]
Core autophagy machinery	BECN1/ATG5/LC3	Autophagy executors; aging-associated epigenetic repression of these genes correlates with reduced flux and compromised mitochondrial quality control in MSCs.	[13,14]
Master transcriptional regulator	TFEB	TFEB drives lysosome/autophagy transcription; promoter methylation represses TFEB in hepatocyte/renal models, and TFEB activation improves aged BMSC function and bone phenotypes.	[15,16,50]
Energy sensing	AMPK	Energy sensor that activates ULK1 and suppresses mTORC1; AMPK-dependent autophagy supports BMSC osteogenesis (e.g., metformin response).	[41,48,53]
Nutrient signaling	mTORC1	Nutrient sensor that suppresses autophagy and MiT/TFE-driven lysosomal programs; relevant to autophagy control in bone-related contexts.	[42,43,53]
Autophagy initiation kinase	ULK1	Autophagy-initiating kinase integrating energy and acetylation inputs (e.g., AMPK signaling; GSK3–TIP60 regulation).	[46,48]
Selective autophagy receptor	p62/SQSTM1	Selective autophagy receptor; p62 accumulation is a proxy for impaired flux and altered turnover of regulatory proteins.	[30,31,32]
Sirtuin hub	SIRT1/SIRT3/SIRT6	NAD+-dependent deacetylases linking redox/energy to chromatin and autophagy; SIRT3 protects BMMSCs from senescence, and SIRT6/SIRT1 are implicated in autophagy–senescence–bone phenotypes.	[28,29,51,52]
Histone deacetylases	HDAC9/HDAC6	HDAC6 supports autophagosome maturation; HDAC9 suppresses autophagy in MSCs and accelerates age-related bone loss, with stress-linked HDAC6/autophagy dysfunction reported in bone MSCs.	[39,40,54]
Lineage fate regulators	RUNX2/SP7 (OSX) vs. PPARγ/C/EBPα	Autophagy and methylation state jointly tune osteogenic (RUNX2/SP7) versus adipogenic (PPARγ/C/EBPα) transcriptional programs in aging MSCs.	[18,55,56]
Methyl-donor metabolism	SAM/one-carbon metabolism	One-carbon metabolism controls SAM availability and methylation; SAM-responsive methylation can suppress autophagy, and SAMS-1 couples SAM status to TFEB-linked programs.	[21,22,23]
Amplification modules	ROS/cGAS–STING/NF-κB/SASP	Autophagy decline promotes mitochondrial ROS and inflammatory pathways (cGAS–STING/NF-κB/SASP), reinforcing adipogenic bias and an anti-osteogenic niche.	[57,58,59,60]

**Table 2:** Non-coding RNAs implicated in MSC aging and lineage commitment.

ncRNA	Reported Target(s)	Functional Implication for MSC Aging/Lineage Bias	References
miR-188	RICTOR; HDAC9	Age-upregulated; targets RICTOR/HDAC9 to drive adipogenic bias; inhibition mitigates age-related bone loss (mouse).	[67]
miR-34a	SIRT1 (p53-associated signaling)	Senescence-associated; targets SIRT1 and amplifies p53 signaling; inhibition alleviates senescence phenotypes (human adipose-derived MSCs).	[68,69]
miR-15b	USP7—KDM6B; autophagy-related pathways	Osteoporosis-associated; targets USP7–KDM6B to suppress autophagy and osteoblast-lineage differentiation (osteoblast-lineage models).	[70]
lncRNA NEAT1	Mitochondrial function; stemness network (indirect)	Elevated in aged BMSCs; disrupts mitochondrial/stemness programs and promotes adipogenesis; knockdown improves bone mass and reduces marrow fat (mouse).	[71]
lncRNA SNHG14	miR-493-5p—MEF2C; autophagy	Acts as a ceRNA to relieve miR-493-5p repression, activate MEF2C/autophagy, and enhance osteogenesis (osteoporosis models).	[72]
lncRNA H19/miR-675	Class II HDACs (HDAC4/5/6)	Restrains BMMSC adipogenesis via class II HDACs.	[73]

## Data Availability

No new data were created or analyzed in this study.

## Supplementary Materials

The following supporting information can be downloaded at https://www.mdpi.com/article/10.3390/biology15030218/s1, Table S1: Representative PTM sites highlighted in the revised text.
