# Supplementary material for: The DNA Methylation–Autophagy Axis: A Driver of MSC Fate Imbalance in Skeletal Aging and Osteoporosis
Source: Biology (Basel). 2026 Jan 24;15(3):218. doi: 10.3390/biology15030218 (PMC12896871; doi:10.3390/biology15030218)
Supplement: Supplementary file 1 [file biology-15-00218-s001.zip › biology-4069772-supplementary.pdf]

Supplementary Table S1. Representative PTM sites highlighted in the revised text

Regulator	Modified protein	Modification	Site(s)(residue)	Functional consequence (context in manuscript)	Supporting references
HDAC6	α-tubulin	Deacetylation	Lys40	Microtubule dynamics and autophagosome–lysosome fusion; excessive activity impairs flux	[38–40]
mTORC1	TFEB	Phosphorylation	Ser142, Ser211	Cytoplasmic retention and repression of autophagy–lysosome transcriptional program	[41, 42]
Calcineurin (PPP3)	TFEB	Dephosphorylation	Ser142/Ser211	Nuclear translocation and induction of autophagy/lysosome genes during nutrient deprivation/oxidative stress	[42, 43, 44]
TIP60 (KAT5)	ULK1	Acetylation	Lys162, Lys606	Acetylation-dependent control of autophagy initiation described for non-MSC systems	[44, 46]
AMPK	ULK1	Phosphorylation	Ser317, Ser777	Activation of autophagy under energetic stress and antagonism of mTOR signaling	[47, 48]
SIRT3	SOD2	Deacetylation	Lys68	Limits ROS accumulation, supports bioenergetics, and indirectly supports autophagy/osteogenesis in aged MSCs	[28, 29]
SIRT1	TFEB	Deacetylation	Lys116	Promotes TFEB nuclear import to enhance autophagy/lysosome gene transcription	[42, 49]
